# Simple Visualized Detection Method of Virulence-Associated Genes of *Vibrio cholerae* by Loop-Mediated Isothermal Amplification

**DOI:** 10.3389/fmicb.2019.02899

**Published:** 2019-12-20

**Authors:** Mengjie Xu, Huiyu Fu, Dailing Chen, Zehuai Shao, Jun Zhu, Walid Q. Alali, Lanming Chen

**Affiliations:** ^1^Key Laboratory of Quality and Safety Risk Assessment for Aquatic Products on Storage and Preservation (Shanghai), China Ministry of Agriculture, College of Food Science and Technology, Shanghai Ocean University, Shanghai, China; ^2^Department of Microbiology, Perelman School of Medicine, University of Pennsylvania, Philadelphia, PA, United States; ^3^Department of Epidemiology and Biostatistics, Faculty of Public Health, Kuwait University, Safat, Kuwait

**Keywords:** *Vibrio cholerae*, virulence-associated genes, loop-mediated isothermal amplification, water, aquatic product

## Abstract

*Vibrio cholerae* is a leading waterborne pathogenic bacterium worldwide. It can cause human cholera that is still pandemic in developing nations. Detection of *V*. *cholerae* contamination in drinking water and aquatic products is imperative for assuring food safety. In this study, a simple, sensitive, specific, and visualized method was developed based on loop-mediated isothermal amplification (LAMP) (designated sssvLAMP) to detect virulence-associated (*ctxA, tcpA*, *hapA*, *mshA*, *pilA*, and *tlh*) and species-specific (*lolB*) genes of *V. cholerae.* Three pairs of oligonucleotide primers (inner, outer, and loop primers) were designed and or synthesized to target each of these genes. The optimal conditions of the sssvLAMP method was determined, and one-step sssvLAMP reaction was performed at 65°C for 40 min. Positive results were simply read by the naked eye via color change (from orange to light green) under the visible light, or by the production of green fluorescence under the UV light (260 nm). The sssvLAMP method was more efficient in detecting 6.50 × 10^1^–6.45 × 10^4^-fold low number of *V. cholerae* cells, and more sensitive in *V. cholerae* genomic DNA (1.36 × 10^–2^-4.42 × 10^–6^ ng/reaction) than polymerase chain reaction (PCR) method. Among 52 strains of *V. cholerae* and 50 strains of non-target species (e.g., other *Vibrios* and common pathogens) examined, the sensitivity and specificity of the sssvLAMP method were 100% for all the target genes. Similar high efficiency of the method was observed when tested with spiked samples of water and aquatic products, as well as human stool specimens. Water from various sources and commonly consumed fish samples were promptly screened by this simple and efficient visualized method and diversified variation in the occurrence of the target genes was observed. *V. cholerae* strains could be mostly detected by the presence of *hapA* and *tlh* alone or in combination with other genes, indicating a variable risk of potentially pathogenic non-O1/O139 strains in edible food products. This novel LAMP method can be a promising tool to address the increasing need of food safety control of aquatic products.

## Introduction

*Vibrio cholerae* can cause cholera, a severe human diarrhoeal disease that can be quickly fatal if untreated and is typically transmitted via contaminated water and person-to-person contact (Baker-Austin et al., 2018). It was estimated that cholera caused roughly 2.9 million cases and 95,000 deaths annually worldwide between 2008 and 2012 (Ali et al., 2015). Cholera is still pandemic in developing nations in recent years such as in Zambia, Tanzania, Mozambique, Somalia, South Sudan, Kenya, and Congo (Kinshasa) in 2018, as well as in Mozambique and Somalia in 2019 (World Health Organization)^1^. *V. cholerae* is reported to harbor a highly conserved species-specific gene *lolB* (Lalitha et al., 2008). Previous studies have indicated that cholerae toxin (CTX) and toxin coregulated pilus (TCP) are major toxic factors of epidemic *V. cholerae* strains of serotypes O1 and O139. Nevertheless, some non-O1/O139 strains lacking the *ctx* and *tcp* genes have been reported to cause sporadic episodes of diarrhea and gastroenteritis (Austin, 2010; Ceccarelli et al., 2015), indicating that other virulence factors exist. Thus, detection of the potential pathogenic non-O1/O139 *V. cholerae* contamination in food is also imperative for assuring food safety.

Previous studies have revealed virulence-associated genes that are present in *V. cholerae* chromosomes (Hacker et al., 1997). For instance, a *hlyA* gene encodes an extracellular pore-forming toxin, produced by biotype El Tor of serogroup O1 and most of the non-O1/O139 strains. The HlyA is known to be associated with multiple virulence-related traits, including the hemolytic activity, lethality, cardiotoxicity, cytotoxicity and enterotoxicity (Benitez and Silva, 2016; Gao et al., 2018). *V. cholerae* produces at least three morphologically distinct types of pili (Hall et al., 1988), including the TCP, mannose-sensitive hemagglutination (MSHA) pilus (Chiavelli et al., 2001; Moorthy and Watnick, 2004), and Type IV-A pilus (Fullner and Mekalanos, 1999; Aagesen and Hase, 2012), which all play important roles in the adaptability and pathogenicity of the bacterium. The *mshBACD* gene cluster is responsible for the structure of MSHA, which is reported to act not only as a receptor of a widespread filamentous bacteriophage facilitating transfer of virulence genes in *V. cholerae* O139 strain (Jouravleva et al., 1998), but also aiding bacterial association with aquatic plankton to support environmental adaptation in non O1/O139 strains (Chiavelli et al., 2001; Moorthy and Watnick, 2004; Gong et al., 2019). The third type of pilus is essential for the colonization of *Vibrio* species in the environment and/or host tissues (Fullner and Mekalanos, 1999). It is encoded by a 5.4-kb *pilABCD* gene cluster, in which the *pilA* gene encodes one of the major subunits of this Type IV-A pilus (Aagesen and Hase, 2012). Among other virulence-associated factors in *V. cholerae* include the *tlh* (Fiore et al., 1997), and *hapA* genes (Datta-Roy et al., 1986), the former encodes a thermolabile hemolysin with phospholipase and lecithinase activities (Fiore et al., 1997), and the latter encodes a hemagglutinin protease involved in *V. cholerae* interaction with aquatic hosts (Halpern et al., 2003).

To date, many methods have been developed for effective detection of O1/O139 *V. cholerae* contamination in food. Compared with the conventional culture-based microbiological detection assays, molecular biology-based methods are more rapid and sensitive, such as PCR (Kumar et al., 2010; Zago et al., 2017), real-time PCR (Garrido-Maestu et al., 2015; Casasola-Rodriguez et al., 2018), multiplex PCR (Bwire et al., 2018; Vu et al., 2018), oligonucleotide array hybridization (Nasrabadi et al., 2017), strand displacement amplification (SDA) (Phillips et al., 2018), rolling circle amplification (RCA) (Osterberg et al., 2014), cross-priming amplification (CPA) (Zhang et al., 2015), and nucleic acid sequence-based amplification (NASBA) (Fykse et al., 2012). Nevertheless, these methods require expensive equipments, which limit their wide application, particularly in on-site testing and large-scale survey. On the other hand, the loop-mediated isothermal amplification (LAMP) technique is an alternative promising tool because of its simplicity, rapidness and suitability for on-site large-scale screening (Notomi et al., 2000; Soli et al., 2013; Engku Nur Syafirah et al., 2018).

The LAMP technique, originally developed by Notomi et al. (2000), can amplify nucleic acids from a single copy to 10^9^ copies at a constant temperature (typically 60–70°C) (Notomi et al., 2000). This one-step reaction method requires just a simple equipment such as a water bath or temperature block. The LAMP-positive amplicons can be confirmed by the gel electrophoresis analysis with fluorescent dsDNA intercalating dyes, e.g., the ethidium bromide (EB) (Almasi et al., 2012) and Synergy Brands (SYBR) Green (Yu et al., 2014; Yang et al., 2018). Utilization of metal indicators, such as hydroxynaphthol blue (HNB) (Soli et al., 2013; Zhong et al., 2018), and magnesium pyrophosphate (Notomi et al., 2000), allows the observation of results with the naked eye. However, the post-amplification detection requires opening of reaction tubes and therefore significantly increases the risk of carry-over contamination (Zanoli and Spoto, 2013). Although the HNB dye can be added prior to the reaction, positive amplicons with color change from violet to sky blue can be variable and imperfect for different observers (Wastling et al., 2010). The drawback of the magnesium pyrophosphate-based detection method is time-dependent and instable. The turbidity generated as a by-product (magnesium pyrophosphate) of DNA amplification of positive samples is stable but just for a short time, which needs to be judged soon after taking out the samples from the water bath (Mori et al., 2001). On the other hand, the use of MnCl_2_-calcein dye has been a more recent approach to circumvent the instability problem of other dyes applied in LAMP-based detection (Fang et al., 2018; Sayad et al., 2018) to develop the sssvLAMP system. Calcein is a metal indicator that yields strong fluorescence by forming complexes with divalent metallic ions, such as Ca^2+^ and Mg^2+^ (Toffaletti and Kirvan, 1980; Fiedoruk-Pogrebniak and Koncki, 2015). As LAMP reaction proceeds in the presence of target DNA, calcein is deprived of Mn^2+^ by newly generated pyrophosphate ion, and instead combines with residual Mg^2+^, producing green fluorescence (Tomita et al., 2008). In contrast, if no amplification occurred, no color change and green fluorescence are observed.

To date, only few studies have been conducted to detect *V. cholerae* genes by the LAMP, i.g., the *ctxA* (Okada et al., 2010), *hlyA* (He et al., 2009), *ompW* (Srisuk et al., 2010), *rtxA* (Tourlousse et al., 2012), *toxR* (Zhang et al., 2015), and *lolB* (Liew et al., 2015). Development of a simple, rapid, specific and sensitive LAMP method for *V. cholerae* detection is very important for food safety control, particularly, as a highly efficient tool for large-scale screening of the bacterial contamination in water and aquatic products.

## Materials and Methods

### Bacterial Strains and Culture Conditions

Bacterial strains and culture media used in this study are listed in Table 1. The thiosulfate citrate bile salt sucrose (TSBS), Luria-Bertani (LB), Tryptic Soy Broth (TSB), and de Man Rogosa Sharpe (MRS) media were purchased from Beijing Land Bridge Technology Co., Ltd., Beijing, China. The Enterobacteria Enrichment (EE), and Brain Heart Infusion (BHI) media were purchased from Qingdao Hope Bio-Technology Co., Ltd., Qingdao, China, while the Marine 2216 was from Becton, Dickinson and Company, United States. *Vibrio* and non-*Vibrio* strains were individually inoculated from laboratory stock at −80°C onto corresponding agar plates, respectively, incubated at 37°C for 16–18 h. Single colonies of each strain was individually streaked into corresponding broth supplemented with 3.0% (pH 8.4–8.5, *Vibrio* strains) and 0.5/1.0% NaCl (pH 7.0–7.2, non-*Vibrio* strains), respectively, and incubated at 37°C for 16–18 h for further analysis.

**TABLE 1:** Bacterial strains and media used in this study.

**Strain**	**Virulence-associated gene type/serotype**	**Source**	**Medium**
*Vibrio cholerae* ATCC39315 (N16961)	Type I/O1 biovar El Tor	ATCC, United States	^∗^
*Vibrio cholerae* GIM 1.449	Type IV/non-O1/O139	GCCC, China	TCBS, LB
*Vibrio cholerae* L10-36	Type III/non-O1/O139	Xu et al., 2019	TCBS, LB
*Vibrio cholerae* Q07-21	Type II/non-O1/O139	Xu et al., 2019	TCBS, LB
*Vibrio cholerae* B02-53	Type V/non-O1/O139	Xu et al., 2019	TCBS, TSB
*Vibrio cholerae* B09-31	Type V/non-O1/O139	Xu et al., 2019	TCBS, TSB
*Vibrio cholerae* J02-10	Type V/non-O1/O139	Xu et al., 2019	TCBS, TSB
*Vibrio cholerae* J06-74	Type V/non-O1/O139	Xu et al., 2019	TCBS, TSB
*Vibrio cholerae* J07-44	Type V/non-O1/O139	Xu et al., 2019	TCBS, TSB
*Vibrio cholerae* L02-13	Type V/non-O1/O139	Xu et al., 2019	TCBS, TSB
*Vibrio cholerae* J09-62	Type V/non-O1/O139	Xu et al., 2019	TCBS, TSB
*Vibrio cholerae* L03-18	Type V/non-O1/O139	Xu et al., 2019	TCBS, TSB
*Vibrio cholerae* L10-04	Type V/non-O1/O139	Xu et al., 2019	TCBS, TSB
*Vibrio cholerae* L10-05	Type IV/non-O1/O139	Xu et al., 2019	TCBS, TSB
*Vibrio cholerae* Q01-39	Type V/non-O1/O139	Xu et al., 2019	TCBS, TSB
*Vibrio cholerae* Q04-43	Type V/non-O1/O139	Xu et al., 2019	TCBS, TSB
*Vibrio cholerae* Q06-10	Type V/non-O1/O139	Xu et al., 2019	TCBS, TSB
*Vibrio cholerae* Q06-66	Type V/non-O1/O139	Xu et al., 2019	TCBS, TSB
*Vibrio cholerae* b06-92	Type IV/non-O1/O139	LS-SHOU, China	TCBS, TSB
*Vibrio cholerae* b10-61	Type IV/non-O1/O139	LS-SHOU, China	TCBS, TSB
*Vibrio cholerae* b10-79	Type IV/non-O1/O139	LS-SHOU, China	TCBS, TSB
*Vibrio cholerae* b11-89	Type IV/non-O1/O139	LS-SHOU, China	TCBS, TSB
*Vibrio cholerae* N03-06	Type IV/non-O1/O139	LS-SHOU, China	TCBS, TSB
*Vibrio cholerae* N04-21	Type V/non-O1/O139	LS-SHOU, China	TCBS, TSB
*Vibrio cholerae* N04-58	Type V/non-O1/O139	LS-SHOU, China	TCBS, TSB
*Vibrio cholerae* N04-70	Type V/non-O1/O139	LS-SHOU, China	TCBS, TSB
*Vibrio cholerae* B05-69	Type II/non-O1/O139	Xu et al., 2019	TCBS, TSB
*Vibrio cholerae* L03-93	Type II/non-O1/O139	Xu et al., 2019	TCBS, TSB
*Vibrio cholerae* J07-29	Type III/non-O1/O139	Xu et al., 2019	TCBS, TSB
*Vibrio cholerae* J07-85	Type III/non-O1/O139	Xu et al., 2019	TCBS, TSB
*Vibrio cholerae* B01-01	Type IV/non-O1/O139	Xu et al., 2019	TCBS, TSB
*Vibrio cholerae* B06-04	Type IV/non-O1/O139	Xu et al., 2019	TCBS, TSB
*Vibrio cholerae* B06-69	Type IV/non-O1/O139	Xu et al., 2019	TCBS, TSB
*Vibrio cholerae* B07-10	Type IV/non-O1/O139	Xu et al., 2019	TCBS, TSB
*Vibrio cholerae* J02-13	Type IV/non-O1/O139	Xu et al., 2019	TCBS, TSB
*Vibrio cholerae* J02-19	Type IV/non-O1/O139	Xu et al., 2019	TCBS, TSB
*Vibrio cholerae* J02-24	Type IV/non-O1/O139	Xu et al., 2019	TCBS, TSB
*Vibrio cholerae* J06-25	Type IV/non-O1/O139	Xu et al., 2019	TCBS, TSB
*Vibrio cholerae* J07-29	Type III/non-O1/O139	Xu et al., 2019	TCBS, TSB
*Vibrio cholerae* L01-06	Type IV/non-O1/O139	Xu et al., 2019	TCBS, TSB
*Vibrio cholerae* L02-20	Type IV/non-O1/O139	Xu et al., 2019	TCBS, TSB
*Vibrio cholerae* L03-10	Type IV/non-O1/O139	Xu et al., 2019	TCBS, TSB
*Vibrio cholerae* L10-06	Type IV/non-O1/O139	Xu et al., 2019	TCBS, TSB
*Vibrio cholerae* Q01-10	Type IV/non-O1/O139	Xu et al., 2019	TCBS, TSB
*Vibrio cholerae* Q01-35	Type IV/non-O1/O139	Xu et al., 2019	TCBS, TSB
*Vibrio cholerae* Q04-55	Type IV/non-O1/O139	Xu et al., 2019	TCBS, TSB
*Vibrio cholerae* Q08-34	Type IV/non-O1/O139	Xu et al., 2019	TCBS, TSB
*Vibrio cholerae* Q10-54	Type IV/non-O1/O139	Xu et al., 2019	TCBS, TSB
*Vibrio cholerae* N05-39	Type IV/non-O1/O139	LS-SHOU, China	TCBS, TSB
*Vibrio cholerae* N05-77	Type IV/non-O1/O139	LS-SHOU, China	TCBS, TSB
*Vibrio cholerae* N06-55	Type IV/non-O1/O139	LS-SHOU, China	TCBS, TSB
*Vibrio cholerae* N08-74	Type IV/non-O1/O139	LS-SHOU, China	TCBS, TSB
*Vibrio alginolyticus* ATCC17749	Type VI	ATCC, United States	TSB
*Vibrio alginolyticus* ATCC33787	Type VI	ATCC, United States	TSB
*Vibrio alginolyticus*	Type VI	DL, China	LB
*Vibrio fluvialis* ATCC33809	Type VI	ATCC, United States	Marine 2216
*Vibrio harvey* ATCC BAA-1117	Type VI	ATCC, United States	Marine 2216
*Vibrio harvey* ATCC33842	Type VI	ATCC, United States	Marine 2216
*Vibrio mimicus* bio-56759	Type VI	Biobw, China	TSB
*Vibrio metschnikovii* ATCC700040	Type VI	ATCC, United States	Marine 2216
*Vibrio parahemolyticus* ATCC17802	Type VI	ATCC, United States	LB
*Vibrio parahemolyticus* ATCC33847	Type VI	ATCC, United States	LB
*Vibrio parahemolyticus* B3-13	Type VI	LS-SHOU, China	TSB
*Vibrio parahemolyticus* B4-10	Type VI	LS-SHOU, China	TSB
*Vibrio parahemolyticus* B5-29	Type VI	LS-SHOU, China	TSB
*Vibrio parahemolyticus* B9-35	Type VI	LS-SHOU, China	TSB
*Vibrio vulnificus* ATCC27562	Type VI	ATCC, United States	TSB
*Vibrio vulnificus*	Type VI	DL, China	LB
*Aeromonas hydrophila* ATCC35654	Type VI	ATCC, United States	TSB
*Aeromonas hydrophila*	Type VI	–	LB
*Enterobacter cloacae* ATCC13047	Type VI	ATCC, United States	TSB
*Enterobacter cloacae*	Type VI	SJAM, China	LB
*Escherichia coli* ATCC8739	Type VI	ATCC, United States	TSB
*Escherichia coli* ATCC25922	Type VI	ATCC, United States	LB
*Escherichia coli* K12	Type VI	IIM, China	TSB
*Enterobacter sakazakii* CMCC45401	Type VI	Biobw, China	TSB
*Klebsiella oxytoca* 0707-27	Type VI	LS-SHOU, China	EE
*Klebsiella pneumoniae* 0717-1	Type VI	LS-SHOU, China	EE
*Klebsiella pneumoniae* 1202	Type VI	LS-SHOU, China	EE
*Klebsiella variicola* 0710-01	Type VI	LS-SHOU, China	EE
*Lactobacillus plantarum* D31	Type VI	LS-SHOU, China	MRS
*Lactobacillus plantarum* T9	Type VI	LS-SHOU, China	MRS
*Lactobacillus casei* K17	Type VI	LS-SHOU, China	MRS
*Listeria monocytogenes* ATCC19115	Type VI	ATCC, United States	BHI
*Pseudomonas aeruginosa* ATCC9027	Type VI	ATCC, United States	TSB
*Pseudomonas aeruginosa* ATCC27853	Type VI	ATCC, United States	TSB
*Salmonella enterica* subsp. Enterica -Leminor et popoff ATCC13312	Type VI	ATCC, United States	TSB
*Salmonella paratyphi*-A CMCC50093	Type VI	GCCC, China	TSB
*Salmonella typhimurium* ATCC15611	Type VI	ATCC, United States	TSB
*Salmonella* spp.	Type VI	–	LB
*Shigella dysenteriae* CMCC51252	Type VI	ATCC, United States	TSB
*Shigella flexneri* CMCC51572	Type VI	GCCC, China	TSB
*Shigella flexneri* ATCC12022	Type VI	ATCC, United States	TSB
*Shigella flexneri* CMCC51574	Type VI	GCCC, China	TSB
*Shigella sonnei* ATCC25931	Type VI	ATCC, United States	TSB
*Shigella sonnet* CMCC51592	Type VI	GCCC, China	TSB
*Staphylococcus aureus* ATCC25923	Type VI	ATCC, United States	TSB
*Staphylococcus aureus* ATCC8095	Type VI	ATCC, United States	TSB
*Staphylococcus aureus* ATCC29213	Type VI	ATCC, United States	TSB
*Staphylococcus aureus* ATCC6538	Type VI	ATCC, United States	TSB
*Staphylococcus aureus* ATCC6538P	Type VI	ATCC, United States	TSB
*Staphylococcus aureus*	Type VI	–	LB

*^∗^, genomic DNA available. −, unknown. ATCC: American Type Culture Collection, United States; DL: Dishui Lake, Shanghai, China; GCCC, Guangdong Culture Collection Center, Guangzhou, China; IIM, Institute of Industrial Microbiology, Shanghai, China; LS-SHOU, Laboratory stock, Shanghai Ocean University, Shanghai, China; SJAM, Shanghai Jiangyang Aquatic Market, Shanghai, China; Virulence-associated gene Type I: *ctxAB*^+^*tcp*^+^*hapA*^+^*mshA*^+^*pilA*^+^*tlh*^+^; Type II: *ctxAB*^–^*tcp*^–^*hapA*^+^*mshA*^+^*pilA*^–^*tlh*^+^; Type III: *ctxAB*^–^*tcp*^–^*hapA*^+^*mshA*^–^*pilA*^+^*tlh*^+^; Type IV: *ctxAB*^–^*tcp*^–^*hapA*^+^*mshA*^–^*pilA*^–^*tlh*^+^; Type V: *ctxAB*^–^*tcp*^–^*hapA*^+^*mshA*^–^*pilA*^–^*tlh*^–^; Type VI: *ctxAB*^–^*tcp*^–^*hapA*^–^*mshA*^–^*pilA*^–^*tlh*^–^.*

### Genomic DNA Preparation

Bacterial genomic DNA was prepared using the TaKaRa MiniBEST Bacterial Genomic DNA Extraction Kit Ver. 3.0 (TaKaRa Biomedical Technology Co., Ltd., Beijing, China) following the manufacturer’s instructions. Extracted DNA samples were analyzed by agarose gel electrophoresis, visualized under short-wavelength UV light (260 nm), and imaged using the UVP EC3 Imaging system (UVP LLC, Upland, CA, United States) as described previously (He et al., 2015). The DNA concentration and purity (A_260_/A_280_) were determined using a multimode microplate reader (Synergy 2, Vermont, United States).

Bacterial genomic DNA was also extracted by a thermal lysis method as described previously (Okada et al., 2010) with minor modifications. Briefly, 1 mL of bacterial cell culture was centrifugated at 12,000 rpm for 5 min, and the cell pellet was resuspended with 1 mL of sterile 1 × phosphate buffer saline (PBS, pH 7.4–7.6, Shanghai Sangon Biological Engineering Technology and Services Co., Ltd., Shanghai, China). The resuspension was then 10-fold serially diluted. Bacterial cells were enumerated via plating appropriate dilutions of cell suspension onto the LB agar plates as described previously (Sun et al., 2014). In parallel, a 1 mL of appropriately diluted resuspension was centrifugated at 12,000 rpm for 5 min, and the cell pellet was heated in 200 μL of sterile ultrapure water at 95°C for 10 min, and then transferred on ice for cooling. After centrifugation at 12,000 rpm for 5 min, the resulting lysis solution was used as DNA template for the detection of *V. cholerae* cells in water.

### Designing LAMP Primers

The primers used in this study were listed in Table 2. The sequences of the target genes of *V. cholerae* were downloaded from the National Center for Biotechnology Information (NCBI) GenBank database^2^, including the *tcpA, hapA*, *mshA*, *pilA*, *tlh*, and *lolB* genes, with GenBank accession Nos. listed in Supplementary Figure S1. Two pairs of inner and outer primers (FIP and BIP, F3 and B3) targeting conserved sequences of each gene was designed using the Primer Explorer Version 5 software^3^ with default parameters by importing DNA sequence of each target gene in a FASTA format, while the pair of the loop primers (LF and LB) were designed using the SnapGene Viewer version 4.1.4 software (GSL Biotech LLC, Chicago, IL, United States) by choosing loop primer sequences based on proper annealing temperatures and sequence locations required by inner and outer primers. The locations of each pair of the newly designed primers were marked in Supplementary Figure S1. The *ctxA* gene was detected with the primers described previously (Yamazaki, 2011). All primers were synthesized by the Sangon (Shanghai, China). Comparative sequence alignments between the newly designed LAMP primers versus target gene sequences were performed using the BioEdit software (Hall, 1999) (Supplementary Figure S1). The virulence-associated genes were amplified from some representative *V. cholerae* strains by PCR reactions, and DNA sequencing was carried out by the Sangon (Shanghai, China). The sequences were submitted to the GenBank with accession Nos. listed in Supplementary Figure S1.

**TABLE 2:** The oligonucleotide primers designed and used in this study.

**Primer**	**Target gene**	**Reaction**	**Sequence (5′ to 3′)**	**Amplicon size (bp)**	**Source**
FIP-hapA	*hapA*	LAMP	CGCTTCCCCTGCGATATCCGGCAGAATTCAGGCCTCGTT		This study
BIP-hapA	*hapA*	LAMP	TATGCGTGGCAATGTCGACTGGCGTAGACCACCGGAGGATT		This study
F3-hapA	*hapA*	LAMP	CGTTAGTGCCCATGAGGTC		This study
B3-hapA	*hapA*	LAMP	CGTGACGGCTGATCGAAAT		This study
LF-hapA	*hapA*	LAMP	AGAATGCTTCGTTAATACCACC		This study
LB-hapA	*hapA*	LAMP	ATTGTCGGCGCGGATATT		This study
F-hapA	*hapA*	PCR	CGTTAGTGCCCATGAGGTC	207	Xu et al., 2019
B-hapA	*hapA*	PCR	CGTGACGGCTGATCGAAAT		Xu et al., 2019
FIP-mshA	*mshA*	LAMP	ACCATTGAAGCCTATGTCAATCCATTTTATTGCGTTGCAATCGTC		This study
BIP-mshA	*mshA*	LAMP	ACGATCTATGTGTCCGTTATACAGCAGTCTGCATAGCAACCGT		This study
F3-mshA	*mshA*	LAMP	CGCTAGATACTTCGAGTGAG		This study
B3-mshA	*mshA*	LAMP	TACCACAAGCAGTTCCAG		This study
LF-mshA	*mshA*	LAMP	TAGCTTGATTACTATTTGTTCCTG		This study
LB-mshA	*mshA*	LAMP	TGCAACCTCTAATAATCCTGCA		This study
F-mshA	*mshA*	PCR	CGCTAGATACTTCGAGTGAG	189	Xu et al., 2019
B-mshA	*mshA*	PCR	TACCACAAGCAGTTCCAG		Xu et al., 2019
FIP-pilA	*pilA*	LAMP	TGATGCTGTTGGGGCAATTACACACGCTTAGGTACTGTTGA		This study
BIP-pilA	*pilA*	LAMP	AATACACATTCGATGCTGGTGTATAATCCATTGGCATCTCTTG		This study
F3-pilA	*pilA*	LAMP	ATCTTACCGTCACCCATGTCT		This study
B3-pilA	*pilA*	LAMP	AGTATCTAGTTCAAAAATCCAAT		This study
LF-pilA	*pilA*	LAMP	CAACAACTGCAGGTACGG		This study
LB-pilA	*pilA*	LAMP	CGGTGGTACTACAAGTCC		This study
F-pilA	*pilA*	PCR	GCGATTGCAATTCCTCAA	227	Xu et al., 2019
B-pilA	*pilA*	PCR	CCTAATGCACCTGATGCT		Xu et al., 2019
FIP-tlh	*tlh*	LAMP	ACAGGGTTTCTGGCTGAACTGTTTCAGGGTTATTGGTGGTCG		This study
BIP-tlh	*tlh*	LAMP	AGAACGCTGTGAAGAGACGCTTGATGATCGGCCGCGAAAT		This study
F3-tlh	*tlh*	LAMP	AAAGAATGCGGATGGTAGCT		This study
B3-tlh	*tlh*	LAMP	TCCGGATCATTGCTCCAAAT		This study
LF-tlh	*tlh*	LAMP	CGTGTAAAACATGTTTTTTTGTCG		This study
LB-tlh	*tlh*	LAMP	AACCACGATTTTGCCGATATTAC		This study
F-tlh	*tlh*	PCR	TGGGAGTGGGCAAAGAAT	274	Xu et al., 2019
B-tlh	*tlh*	PCR	AAAGGCTATCGCCAAACG		Xu et al., 2019
FIP-ctxA	*ctxA*	LAMP	TCTGTCCTCTTGGCATAAGACGCAGATTCTAGACCT CCTG		Yamazaki, 2011
BIP-ctxA	*ctxA*	LAMP	TCAACCTTTATGATCATGCAAGAGGCTCAAACTAATTGAGGTGGAA		Yamazaki, 2011
F3-ctxA	*ctxA*	LAMP	GCAAATGATGATAAGTTATATCGG		Yamazaki, 2011
B3-ctxA	*ctxA*	LAMP	GMCCAGACAATATAGTTTGACC		Yamazaki, 2011
LF-ctxA	*ctxA*	LAMP	CACCTGACTGCTTTATTTCA		Yamazaki, 2011
LB-ctxA	*ctxA*	LAMP	AACTCAGACGGGATTTGTTAGG		Yamazaki, 2011
F-ctxAB	*ctxA*	PCR	TGAAATAAAGCAGTCAGGTG	778	McGrath et al., 2006
R-ctxAB	*ctxA*	PCR	GGTATTCTGCACACAAATCAG		McGrath et al., 2006
FIP-tcpA	*tcpA*	LAMP	CGCTTGTAACCAAAGTCTTACATTG-TAAAGCATTCGCAATTACAGT		This study
BIP-tcpA	*tcpA*	LAMP	CCATTTATCAACGTGAAAGAAGGTG-GACACTCGTTTCGAAATCAC		This study
F3-tcpA	*tcpA*	LAMP	TGCTTGGGTCAAGCCACC		This study
B3-tcpA	*tcpA*	LAMP	TCGCTGCTGTCGCTGATC		This study
LF-tcpA	*tcpA*	LAMP	TTTCCACGAAACTCTGCA		This study
LB-tcpA	*tcpA*	LAMP	ACTTAATTACGCCAGCGC		This study
F-tcpA	*tcpA*	PCR	ATGCAATTATTAAAACAGCTTTTTAAG	675	Kumar et al., 2010
R-tcpA	*tcpA*	PCR	TTAGCTGTTACCAAATGCAACAG		Kumar et al., 2010
FIP-lolB	*lolB*	LAMP	CGACCTGTAAGTTCAGCACGGTTCAATGGCAAAAAAGCCCAC		This study
BIP-lolB	*lolB*	LAMP	GTGCGCGGGTCGAAACTTATGAAATTGCGGATCAGGCTTTGT		This study
F3-lolB	*lolB*	LAMP	TCAGCGACAATCGTTCAACT		This study
B3-lolB	*lolB*	LAMP	TCAAGCTGTTCAACGGGAAT		This study
LF-lolB	*lolB*	LAMP	ACTCTCACTGCGTTTAAGCAAT		This study
LB-lolB	*lolB*	LAMP	CAAATCTACCGCGACCAAGATG		This study
VHMF	*lolB*	PCR	TGGGAGCAGCGTCCATTGTG	516	Lalitha et al., 2008
VHA-AS5	*lolB*	PCR	CAATCACACCAAGTCACTC		Lalitha et al., 2008

### Preparation of MnCl_2_-Calcein Dye Stock Solution

Calcein (Sigma, St. Louis, MO, United States) was first dissolved with 1 M NaOH (Analytical Reagent, Sinopharm Chemical Reagent Co., Ltd, Shanghai, China), and 6.5 mM calcein solution was prepared with ultrapure water. MnCl_2_⋅4H_2_O (Sangon, Shanghai, China) was used to prepare 130 mM MnCl_2_ solution. Then a stock solution consisting of 1.30 mM calcein and 15.60 mM MnCl_2_ was prepared and stored at −20°C.

### Optimization of Reaction Parameters of the sssvLAMP Method

The initial LAMP reaction solution was prepared according to the method described previously (Srisuk et al., 2010; Yamazaki, 2011) with minor modifications. A 25 μL of LAMP reaction solution contained 1.6 μM of each of the inner primers (FIP and BIP), 0.2 μM of each of the outer primers (F3 and B3), and 0.8 μM of each of the loop primers (LF and LB), 1× Thermopol buffer (pH 8.8, contains 20 mM Tris-HCl, 10 mM (NH_4_)_2_SO_4_, 10 mM KCl, 2 mM MgSO_4_, 0.10% Triton^®^ X-100, New England Biolabs, Beverly, MA, United States), 0.8 M betaine (Sigma, St. Louis, MO, United States), 1.4 mM of each dNTP (TaKaRa, China), 6 mM MgSO_4_ (New England Biolabs), 12 U *Bst* DNA polymerase (New England Biolabs), and 2 μL of DNA template. Finally, 1 μL of the MnCl_2_-calcein stock solution was added to the reaction solution, in which final concentrations of Mn^2+^ and calcein were 600 and 50 μM, respectively. A negative control was prepared using DNase/RNase-free deionized water (Tiangen Biotech Co., Ltd., Beijing, China) instead of bacterial culture or DNA template.

The reaction parameters were optimized, including different concentrations of the outer primers (0.05–0.40 μM), inner primers (0.4–2.4 μM), loop primers (0.2–1.2 μM), Mg^2+^ (4.0–14.0 mM), dNTP (1.0–2.0 mM), and *Bst* DNA polymerase (2–16 Unit) per reaction, as well as different reaction temperature (50–70°C) and reaction time (30–80 min) (Table 3). The LAMP was terminated at 80°C for 10 min. Additionally, the function of loop primers in the reaction system was also investigated. Some target genes were used in the optimization of reaction parameters.

**TABLE 3:** Optimization of reaction parameters of the sssvLAMP method.

**Parameter^∗^**	**Concentration**	**Constant reaction factors^∗^**	**Green fluorescence**	**Ladder-like DNA pattern**	**Optimized parameter**
Outer primers		1.60 μM inner primers, 0.80 μM loop primers, 1.40 mM dNTP, 6 mM MgSO_4_, 12 Unit *Bst* DNA polymerase, 65°C, 60 min			0.05 μM
	0.00 μM		−	−	
	0.05 μM		+++	++	
	0.10 μM		+++	++	
	0.15 μM		++	++	
	0.20 μM		++	++	
	0.25 μM		++	++	
	0.30 μM		++	++	
	0.35 μM		++	++	
	0.40 μM		++	++	
Inner primers		0.05 μM outer primers, 0.80 μM loop primers, 1.40 mM dNTP, 6 mM MgSO_4_, 12 Unit *Bst* DNA polymerase, 65°C, 60 min			1.60 μM
	0.00 μM		−	−	
	0.40 μM		+	+	
	0.80 μM		++	++	
	1.20 μM		++	++	
	1.60 μM		+++	+++	
	2.00 μM		++	++	
	2.40 μM		++	++	
Loop primers		1.60 μM inner primers, 0.05 μM outer primers, 1.40 mM dNTP, 6 mM MgSO_4_, 12 Unit *Bst* DNA polymerase, 65°C, 60 min			0.20 μM
	0.00 μM		+	+	
	0.20 μM		+++	+++	
	0.40 μM		+++	+++	
	0.60 μM		++	++	
	0.80 μM		++	++	
	1.00 μM		++	++	
	1.20 μM		++	++	
Mg^2+^		1.60 μM inner primers, 0.05 μM outer primers, 0.20 μM loop primers, 1.40 mM dNTP, 12 Unit *Bst* DNA polymerase, 65°C, 60 min			6.00 mM
	4.00 mM		–	+	
	6.00 mM		+	++	
	8.00 mM		+++	++	
	10.00 mM		+++	++	
	12.00 mM		++	++	
	14.00 mM		–	+	
dNTP		1.60 μM inner primers, 0.05 μM outer primers, 0.20 μM loop primers, 6 mM MgSO_4_, 12 Unit *Bst* DNA polymerase, 65°C, 60 min			1.00 mM
	1.00 mM		++	++	
	1.20 mM		++	++	
	1.40 mM		++	++	
	1.60 mM		++	++	
	1.80 mM		++	++	
	2.00 mM		+ +	++	
*Bst* DNA polymerase		1.60 μM inner primers, 0.05 μM outer primers, 0.20 μM loop primers, 1.00 mM dNTP, 6 mM MgSO_4_, 65°C, 60 min			8 Unit
	2 Unit		-	+	
	4 Unit		+	+	
	6 Unit		+	+	
	7 Unit		++	++	
	8 Unit		+++	+++	
	9 Unit		+++	+++	
	10 Unit		++	++	
	12 Unit		++	++	
	14 Unit		+	+	
	16 Unit		+	+	
Reaction temperature		1.60 μM inner primers, 0.05 μM outer primers, 0.20 μM loop primers, 1.00 mM dNTP, 6 mM MgSO_4_, 8 Unit *Bst* DNA polymerase, 60 min			65°C
	50°C		+	+	
	55°C		+	+	
	60°C		++	++	
	65°C		+++	+++	
	68°C		–	+	
	70°C		–	–	
Reaction time		1.60 μM inner primers, 0.05 μM outer primers, 0.20 μM loop primers, 1.00 mM dNTP, 6 mM MgSO_4_, 8 Unit *Bst* DNA polymerase, 65°C			40 min
	30 min		+	+	
	40 min		++	++	
	50 min		+++	+++	
	60 min		+++	+++	
	70 min		–	–	
	80 min		–	–	

*+, presence; −, absence. ^∗^: all the LAMP reactions contained 0.80 M betaine, 600 μM Mn^2+^, and 50 μM calcein.*

### Confirmation of the LAMP Results

Positive and negative results were confirmed by observing the color change of the sssvLAMP reaction solutions with the naked eye under the visible light, or under the UV transilluminator (302 nm) on a blue background, if needed. A light green color or bright fluorescence is typical characteristics of all positive reactions, while an original orange color or no fluorescence for negative results. Also, the LAMP products were verified by 2.0% agarose gel electrophoresis analysis, which forms ladder-like DNA patterns (Tomita et al., 2008). The gels were visualized and recorded using a UVP EC3 Imaging system as described above.

### Determination of Specificity and Sensitivity of the sssvLAMP Method

The inclusivity (determined as 100% of positive detection of target strains) of the sssvLAMP method was examined with 52 *V. cholerae* strains (Table 1), including pandemic (i.g., ATCC39315/N16961), and non-pandemic strains. Among these strains, *V. cholerae* GIM 1.449 is widely used as a reference non-O1/O139 strain in China. Virulence-associated gene types of non-pandemic strains were confirmed by PCR assays (Xu et al., 2019). The exclusivity (determined as 100% of negative detection of non-target strains) of the method was tested with 50 bacterial strains, including closely related *Vibrio* species (*n* = 16), and non-*Vibrio* species (*n* = 34). It contained common bacterial pathogens, e.g., *Vibrio alginolyticus, Vibrio fluvialis, Vibrio harvey, Vibrio metschnikovii, Vibrio mimicus, Vibrio parahemolyticus, Vibrio vulnificus*, as well as *Aeromonas hydrophila*, *Escherichia coli*, *Enterobacter cloacae*, *Enterobacter sakazakii*, *Klebsiella pneumoniae*, *Listeria monocytogenes, Salmonella paratyphi*, and *Staphylococcus aureus* (Table 1). Genomic DNA was individually extracted from these bacteria as described above and diluted serially with the DNase/RNase-free deionized water as DNA templates.

The sensitivity of the sssvLAMP method was evaluated for each targeted gene in the 52 *V. cholerae* strains (Table 1). Cell culture and genomic DNA samples of these strains were used in the evaluation. For the detection of *V. cholerae* cells, overnight cell cultures of *V. cholerae* strains were individually inoculated (1%, v/v) into fresh media (Table 1) and incubated at 37°C, and bacterial cells grown to log-phase were harvested by centrifugation, resuspended, diluted and enumerated as described above. For instance, 9.3 × 10^7^–9.3 colony forming units (CFU)/mL, and 1.44 × 10^6^–1.44 CFU/mL of *V. cholerae* GIM 1.449 cells were used for the detection of the *hapA*, and *tlh* genes, respectively. The 1.09 × 10^8^–1.09 CFU/mL of *V. cholerae* Q07-21 cells were tested for the *mshA* gene, while 1.28 × 10^8^–12.8 CFU/mL of *V. cholerae* L10-36 cells were for the *pilA* gene. For the detection of *V. cholerae* genomic DNA, for instance, 1.67 × 10^1^–1.67 × 10^–9^ ng/μL of *V. cholerae* GIM 1.449 genomic DNA samples was used for the detection of the *hapA* gene, while 1.41 × 10^1^–1.41 × 10^–9^ ng/μL of *V. cholerae* Q07-21, 1.31 × 10^1^–1.31 × 10^–9^ ng/μL of *V. cholerae* L10-36, and 1.48 × 10^1^–1.48 × 10^–9^ ng/μL of *V. cholerae* GIM 1.449 genomic DNA samples were tested for the target genes *mshA*, *pilA*, and *tlh* by the sssvLAMP method, respectively. The last dilution of genomic DNA or cell culture samples, which was tested positive for the target gene by the sssvLAMP method, was considered as the limit of detection (LOD) in this study.

### Determination of Sensitivity of the sssvLAMP Method for Spiked Fish, Crustaceans and Shellfish Samples, as well as Human Stool Specimens

Fresh fish (*Parabramis pekinensis*), crustaceans (*Litopenaeus vannamei*), and shellfish (*Perna viridis*) were sampled from a local food market (30°53′7.16′′N, 121°54′48.39′′E) in Shanghai, China. Fresh meat of the aquatic products without skin was collected with sterile surgical scalpels, and then homogenated (Yamazaki, 2011). The resulting homogenates were plated onto the TCBS agar plates. Only the sample homogenates that were detected negative for *V. cholerae* and the virulence-associated genes were used in the following spiked experiments.

The spiked fish, crustaceans and shellfish samples, as well as human stool specimens were prepared according to the method described previously (Yamazaki, 2011) with minor modifications. Briefly, 1 g (wet weight) of fresh sample was individually added into 9 mL alkaline peptone water (APW, pH 8.5, 3% NaCl, Land Bridge, Beijing, China) and homogenized thoroughly. *V. cholerae* strains GIM1.449, Q07-21, and L10-36 were individually inoculated into 5 mL TSB (pH 8.5, 3% NaCl) broth and incubated at 37°C for 16–18 h. Serial 10-fold dilutions of *V. cholerae* culture were prepared, and bacterial cells were calculated by plating counting method as described above, and meanwhile, a 100 μL of each dilution was spiked into 900 μL of the fresh homogenate and mixed well. Then, two microliters of 10-fold dilution of the mixture was used for the sssvLAMP method targeting the *hapA*, *mshA*, *pilA*, and *tlh* genes, while one microliter was used for the PCR assay.

### PCR Assay

Oligonucleotide primers used for the PCR assay were listed in Table 2. A 20 μL of the PCR reaction solution contained 8 μL of DNase/RNase-Free Deionized Water (Tiangen Beijing, China), 10 μL of 2 × Taq Master Mix (Novoprotein Technology Co., Ltd., Shanghai, China), 0.5 μL of each primer and 1 μL of bacterial culture or genomic DNA template. PCR reaction was performed for 30 cycles, each of which consisted of denaturation at 94°C for 1 min, annealing at 52–62°C for 1 min and extension at 72°C for 1 min. The annealing temperatures and elongation times were based on melting temperatures of primer pairs and the predicted lengths of PCR products. PCR reactions were performed in a Mastercycler^®^ pro PCR thermal cycler (Eppendorf, Hamburg, Germany). Amplicons were analyzed by agarose gel electrophoresis, then visualized and recorded as described above.

### Sample Collection and Analysis by the sssvLAMP Method

Water samples were collected from various sources in June of 2019 in Shanghai, China, including mineral water, spring water, tap water, river water, lake water, and sea water along the East China Sea (Table 4). The latter three types of water samples (*n* = 3 per type) were collected from the surface water layers (<30 cm) as described previously (He et al., 2015). The commonly consumed mineral water (*n* = 3), and spring water (*n* = 3) samples were purchased from local food markets in Shanghai, while the tap water samples (*n* = 3) were collected from the tap water system available throughout the city. Commonly consumed freshwater fish (*Aristichthys nobilis*, *Carassius auratus*, *Ctenopharyngodon idellus*, and *P. pekinensis*) samples (*n* = 3 per fish species, >500 g/sample) were collected from the local food markets as described above. Health huaman stool samples were provided by M. Yang in Shanghai Ocean University. All samples were maintained at 4°C and analyzed immediately after transported to the laboratory in Shanghai Ocean University, Shanghai, China. Bacterial cells of each 500 mL of water samples (>1.5 L/sample) were filtered through polycarbonate membranes with 0.22-μm pore size (47 mm diameter, Millipore, Corcaigh, Ireland). Consequently, each membrane was washed with 10 mL of sterile 1 × PBS. After centrifugation, bacterial cell pellet was resuspended with 1 mL of the 1 × PBS, and 2 μL of which was used as the DNA template for the sssvLAMP as described above. Fish meat and intestinal samples, as well as human stool specimens were homogenated according to the methods described above. All tests in this study were conducted in triplicate.

**TABLE 4:** The genetic diversity of virulence-associated genes of *V. cholerae* in water and fish samples by the sssvLAMP method.

**Sample**	**Number of sample**	**Virulence-associated gene type**	**Number of sample**
**Water sample**			
Mineral water	3	Type VI	3
Spring water	3	Type VI	3
Tap water	3	Type VI	3
River water	3	Type VI	3
Lake water	3	Type VI	3
Sea water	3	Type VI	3
**Fish meat sample**			
*A. nobilis*	3	Type VI	3
*C. auratus*	3	Type VI	3
*C. idellus*	3	Type VI	3
*P. pekinensis*	3	Type VI	3
**Fish intestinal sample**			
*A. nobilis*	3	Type IV	3
*C. auratus*	3	Type V	2
		Type VI	1
*C. idellus*	3	Type VI	3
*P. pekinensis*	3	Type VII	1
		Type V	2

*−, absence; +, presence. Virulence-associated gene Type VII: *ctxAB*^–^*tcp*^–^*hapA*^+^*mshA*^–^*pilA*^+^*tlh*^–^.*

## Results

### Reaction Parameters Optimized for the sssvLAMP Method

#### Optimal Concentration of Outer Primers

**FIGURE 1:**
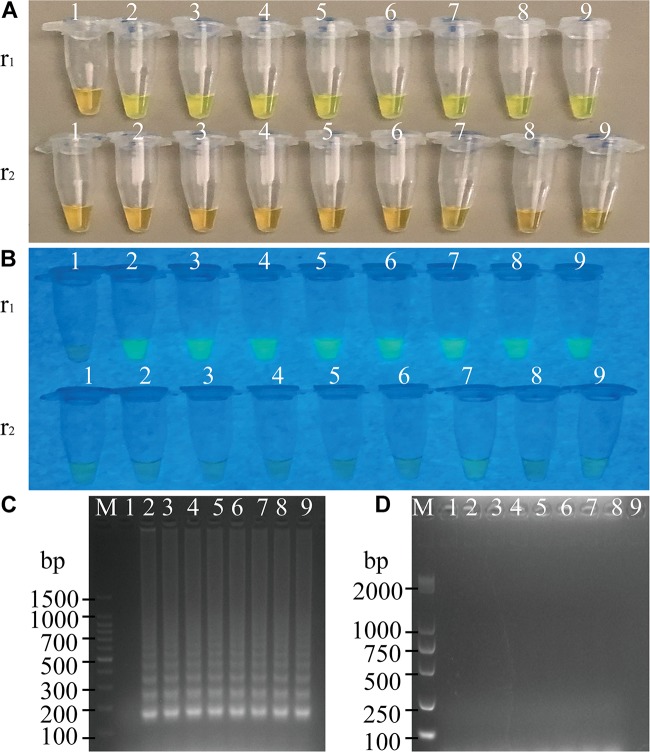
Optimization of the outer primers (F3 and B3) of the sssvLAMP method. **(A–D)** The results observed by the naked eye under the visible light **(A)** and the UV light (302 nm) **(B)**, and verified by 2% agarose gel electrophoresis analysis (**C:** positive results; **D:** negative results). r1 and r2: with and without DNA templates, respectively. Lane M: DNA molecular weight marker (100 bp). Tubes/Lanes 1–9: containing 0.00, 0.05, 0.10, 0.15, 0.20, 0.25, 0.30, 0.35, and 0.40 μM of the F3 and B3 primers, respectively.

To determine the optimal concentration of outer primers for the sssvLAMP method, different concentrations of the F3 and B3 primers (0.05–0.40 μM) were evaluated, while 1.6 μM of inner primers, and 0.8 μM of loop primers were set in reaction systems according to previous reports. The results are illustrated in Figure 1 and Table 3. The color of all the reaction solutions containing 0.05–0.40 μM of the outer primers were observed to change from the original orange to light green by the naked eye under the visible light (Figure 1A, r1: tubes 2–9), and also to display bright green fluorescence under the UV light (Figure 1B, r1: tubes 2–9), which indicated positive amplicons of the target gene (*mshA*) in these reaction tubes. Moreover, no obvious difference in color or fluorescence was found among these positive reactions. These results were confirmed by the agarose gel electrophoresis analysis, which showed characteristic ladder-like DNA patterns (Figure 1C, lanes 2–9). In contrast, the reaction solution without the outer primers was in orange (Figure 1A, tube 1), and showed no green fluorescence under the UV light (Figure 1B, tube 1), indicating no amplification of the target gene. The negative result was also confirmed by the electrophoresis analysis, on which no characteristic ladder-like DNA pattern was observed (Figure 1C, lane 1). Thus, for a cost-effective purpose, the 0.05 μM of the outer primers were chosen for the sssvLAMP method in the further analysis.

#### Optimal Concentration of Inner Primers

Different concentrations (0.4, 0.8, 1.2, 1.6, 2.0, and 2.4 μM) of the inner primers were examined in the sssvLAMP reaction system (Table 3). The target gene was amplified from all reaction solutions supplemented with the FIP and BIP primers, based on the observation of the color change and green fluorescence under the visible light and UV light, respectively (Figures not shown). Nevertheless, the amplification with 0.4 μM of the inner primers yielded weaker ladder-like DNA patterns than those with the other concentrations, as confirmed by the agarose gel electrophoresis analysis (Figures not shown). Moreover, the concentration of 1.6 μM primers produced the strongest DNA bands (Figures not shown). Therefore, the 1.6 μM of the inner primers was chosen for the sssvLAMP method in the further analysis.

#### Optimal Concentration of Loop Primers

As summarized in Table 3, different concentrations (0.2–1.2 μM) of the loop primers were evaluated for the sssvLAMP method, in which 0.05 μM of the outer primers and 1.6 μM of the inner primers were involved. The target gene was amplified from all the reaction tubes containing the 0.2–1.2 μM of the LF and LB primers, showing the color change, production of bright green fluorescence and characteristic ladder-like DNA patterns (Figures not shown). Moreover, no observed difference was found among the positive reactions. Thus, the 0.2 μM of outer primers were chosen for the sssvLAMP method. The optimal proportion among the outer, inner, and loop primers was 1:32:4 (μM/μM/μM).

#### Optimal Concentration of Mg^2+^

The optimal concentration of Mg^2+^ for the sssvLAMP method was determined, and the results were shown in Table 3. The color of reaction solutions containing 6.0–12.0 mM of Mg^2+^ was all observed to change from orange to light green, which indicated positive amplification of the target gene (Figures not shown). These results were confirmed by the agarose gel electrophoresis analysis, which showed characteristic ladder-like DNA patterns (Figures not shown). In contrast, no color change was observed in the reaction solutions containing 4.0 and 14.0 mM of Mg^2+^, respectively, while only weak ladder-like DNA pattern was in the tube with 14.0 mM Mg^2+^ (Figures not shown). Thus, the concentration of 6 mM Mg^2+^ was chosen for the sssvLAMP method.

#### Optimal Concentration of dNTP

As presented in Table 3, the optimal concentration of dNTP was examined for the sssvLAMP method. All the concentrations of dNTP (1.0–2.0 mM) led to the same color change typical for positive results (Figures not shown), consistent with the results yielded from the electrophoresis analysis (Figures not shown). Thus, the minimum concentration of dNTP (1.0 mM) was chosen in the further analysis.

#### Optimal Unit of Bst DNA Polymerase

As shown in Table 3, *Bst* DNA polymerase was also tested in the sssvLAMP system. A weak amplification of the target gene was observed in the reaction solution containing 2 Unit of *Bst* DNA polymerase (Figures not shown). All the other reaction solutions with 4–16 Unit enzyme were in green, indicating positive amplicons in these reaction tubes (Figures not shown). Moreover, the reaction tube with 8 U enzyme showed the strongest ladder-like DNA pattern (Figures not shown). Therefore, the 8 U of *Bst* DNA polymerase was chosen for the sssvLAMP method.

#### Optimal Reaction Temperature

To determine the optimal reaction temperature for the sssvLAMP method, different temperatures (50–70°C) were tested, and the results were presented in Table 3. The typical color change was observed in the reaction tubes when incubated at 50, 55, 60, and 65°C, respectively, whereas no color change was found in the tube incubated at 68 and 70°C (Figures not shown). The results were consistant with those by agarose gel electrophoresis analysis, except that only weak ladder-like DNA pattern was observed in the reaction tube at 68°C (Figures not shown). The sssvLAMP appeared to be completely inhibited at 70°C. The reaction temperature of 65°C was chosen for the further analysis, given the manufacturer’s suggestion for the *Bst* DNA polymerase.

#### Optimal Reaction Time and Utility of the Loop Primers

Variation of reaction times (30–80 min) was also tested, and the results were shown in Table 3. Without the loop primers, a light green color was observed in the tube after the LAMP reaction was performed for 30 min (Figures not shown). However, the sssvLAMP produced bright green fluorescence of good intensity for positive reaction tubes after incubated for 40, 50, 60, 70, and 80 min, respectively (Figures not shown), in congruence with the visualization of strong bands of DNA amplicons by gel electrophoresis was observed, and there was no obvious difference of fluorescence intensity due to variable reaction time (40–80 min).

**FIGURE 2:**
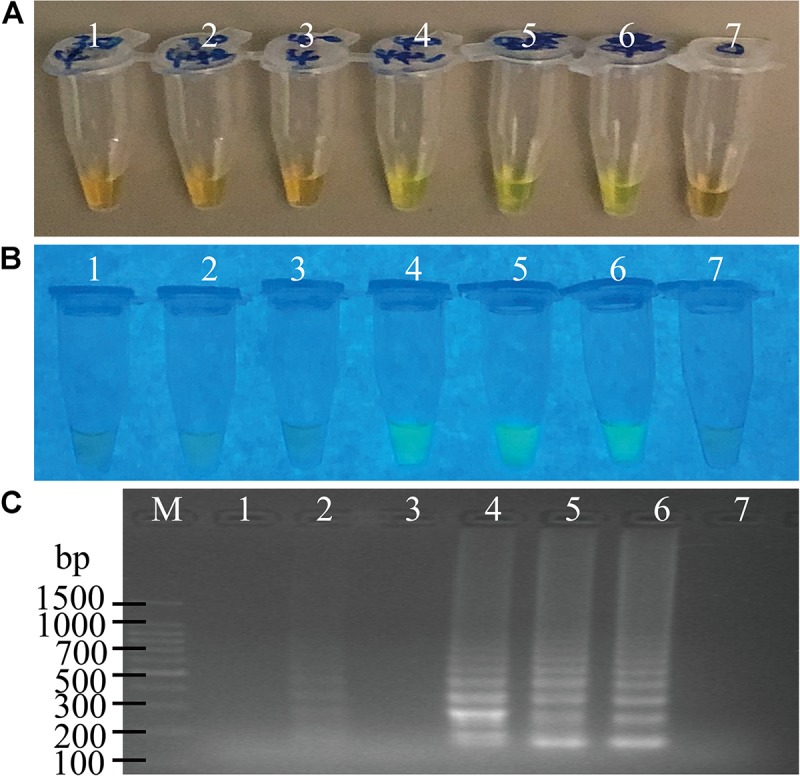
The function of the loop primers (LF and LB) in the sssvLAMP method. **(A–C)** The results observed by the naked eye under the visible light **(A)** and the UV light (302 nm) **(B)**, and verified by 2% agarose gel electrophoresis analysis **(C)**. Lane M: DNA molecular weight Marker (100 bp). Tubes/Lanes 2, 4, 6: reaction mixture with the loop primers at 65°C for 30, 40, 50 min, respectively, while Tubes/Lanes 1, 3, 5: reaction mixture without the loop primers. Tube/Lane 7: negative control.

The usefulness of reaction time reduction by addition of the loop primers was observed. As illustrated in Figure 2, the reaction solution without the loop primers turned light green in color after the reaction was performed for 50 min (Figures 2A,B, tube 5), but those containing the LF and LB primers (0.2 μM) turned light green at 40 min (Figures 2A,B, tube 4). The results by the electrophoresis analysis indicated that there was a weak amplification in the reaction tube with the loop primers at 30 min (Figure 2C, lane 2). These results indicated that the loop primers increased the amplification efficiency and shorten reaction time in the sssvLAMP method.

Taken together, the optimal parameters of the sssvLAMP method included 1.6 μM of FIP and BIP primers, 0.05 μM of F3 and B3 primers, 0.20 μM of LF and LB primers, 6 mM Mg^2+^, 1.0 mM dNTP, and 8 U of *Bst* DNA polymerase. The LAMP reaction was performed at 65°C for 40 min with 0.2 μM of the loop primers (Table 3).

### Specificity of the sssvLAMP Method

**FIGURE 3:**
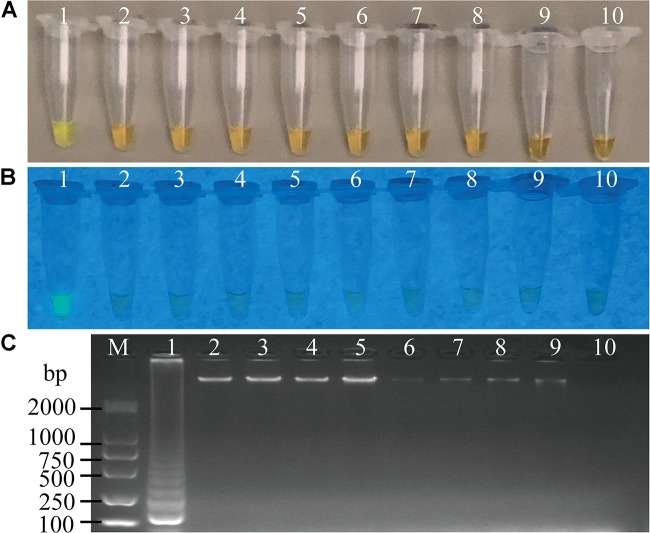
Specificity of the sssvLAMP method for the detection of the *hapA* gene in some bacterial strains. **(A–C)** The results observed by the naked eye under the visible light **(A)** and the UV light (302 nm) **(B)**, and verified by 2% agarose gel electrophoresis analysis **(C)**. Lane M: DNA molecular weight Marker (100 bp). Tubes/Lanes 1–10: containing genomic DNA of *V. cholerae* GIM 1.449, 216.79 ng/μL; *V. parahemolyticus* ATCC 33847, 226.93 ng/μL; *V. vulnificus*, 421.14 ng/μL; *V. alginolyticus*, 225.90 ng/μL; *E. coli* ATCC25922, 94.45 ng/μL; *S. aureus*, 94.09 ng/μL; *A. hydrophila*, 10.18 ng/μL; *Salmonella* spp., 78.54 ng/μL; *E. cloacae*, 132.28 ng/μL; negative control, respectively.

To evaluate specificity of the sssvLAMP method developed in this study, we firstly examined the inclusivity of the method with the 52 *V. cholerae* strains (Table 1). The results indicated that all *V. cholerae* strains were detected positive for the species-specific *lolB* gene. Nevertheless, diversified variation in the occurrence of virulence-associated genes (Types I to VII) was observed. *V. cholerae* strains were mostly detected by the presence of *hapA* and *tlh* alone or in combination with other genes (*mshA*, *pilA*), but the absence of the *ctxA* and *tcpA* genes, except that *V. cholerae* ATCC39315 (N16961) was detected positive for the latter two toxigenic genes (Figures not shown) (Table 1). Consequentially, the exclusivity of the sssvLAMP method was tested with the 50 bacterial strains (Table 1). No positive amplification targeting any of the target genes was recorded in the reaction tubes containing DNA templates of the closely related *Vibrio* species tested (*n* = 16), including *V. fluvialis, V. harvey, V. metschnikovii, V. mimicus, V. parahemolyticus*, and *V. vulnificus*, based on the observed origin color, no green fluorescence and no characteristic ladder-like DNA patterns (e.g., Figures 3, 7). Also, no positive amplification was observed from non-*Vibrio* species (*n* = 34), including the common bacterial pathogens tested, indicating high specificity of the sssvLAMP method targeting the virulence-associated genes of *V. cholerae*.

### LOD of the sssvLAMP Method for Cell Culture and Genomic DNA of *V. cholerae* Strains

**FIGURE 4:**
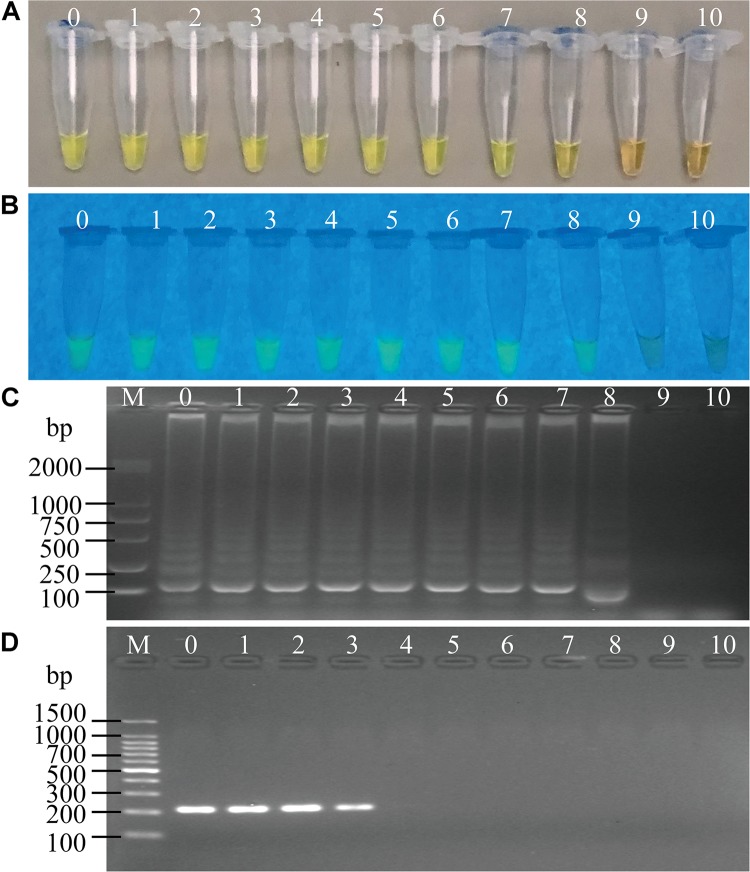
Sensitivity of the sssvLAMP method for the detection of the *hapA* gene of *V. cholerae* cell culture. **(A–C)** The results observed by the naked eye under the visible light **(A)** and the UV light (302 nm) **(B)**, and verified by 2% agarose gel electrophoresis analysis **(C)** by the sssvLAMP method. **D:** the results by the PCR assay. Lane M: DNA molecular weight Marker (100 bp). Tubes/Lanes 0–9: containing 9.3 × 10^7^–9.3 × 10^–2^ CFU/mL of *V. cholerae* GIM 1.449 cells, respectively; 10: negative control.

Sensitivity of the sssvLAMP method was determined for the detection of the 52 *V. cholerae* strains (Table 5). For instance, for the target gene *hapA*, *V. cholerae* GIM 1.449 cells ranged from 9.3 × 10^7^ to 9.3 CFU/mL were tested in the reaction tubes. Cell cultures (5.6 × 10^8^–1.25 CFU/mL) of the other 23 *V. cholerae* strains carrying the *hapA* genes were examined as well. The positive results with the typical color change, production of the bright green fluorescence, and forming of characteristic ladder-like DNA pattern showed that the sensitivity ranged from 1.3 × 10^–1^ to 92 CFU/reaction for 91.3% of the tested *V. cholerae* strains (e.g., Figure 4). Similarly, for the target gene *mshA*, the 3 *V. cholerae* strains (*mshA*^+^) were tested with 1.09 × 10^8^–1.17 CFU/mL cells, and the results indicated that the LOD ranged from 1.5 × 10^–1^ to 28 CFU/reaction (Figures not shown) (Table 5). Likewise, the LOD values for the *pilA* and *tlh* genes ranged from 1.1 × 10^–1^ to 33 CFU/reaction (100% strains tested) and from 1.7 × 10^–1^ to 70 CFU/reaction (87% strains tested), respectively, when cell cultures ranged from 1.28 × 10^8^ to 1.04 CFU/mL of 3 *V. cholerae* (*pilA*^+^) and from 3.5 × 10^7^ to 1.01 CFU/mL of 23 *V. cholerae* (*tlh*^+^) strains were tested, respectively (Figures not shown) (Table 5).

**TABLE 5:** Sensitivity of the sssvLAMP method for the detection of genomic DNA and cell cultures of *V. cholerae* strains.

***V. cholerae* strain**	**Target gene**	**Genomic DNA dilutions (ng/μl)**	**LOD^∗^/reaction (ng)**	**Cell culture dilutions (CFU/mL)**	**LOD^∗^/reaction (CFU)**
ATCC39315	*hapA*	48.11-4.81 × 10^–8^	9.62 × 10^–4^	–	–
GIM 1.449	*hapA*	16.68-1.67 × 10^–9^	4.42 × 10^–6^	9.30 × 10^7^-9.30	2.4 × 10^–2^
B02-53	*hapA*	14.77-1.48 × 10^–8^	2.95 × 10^–5^	1.68 × 10^7^-1.68	3.4
B09-31	*hapA*	23.93-2.39 × 10^–8^	4.79 × 10^–4^	1.42 × 10^7^-1.42	2.8 × 10^1^
J02-10	*hapA*	21.23-2.12 × 10^–8^	4.25 × 10^–6^	3.90 × 10^6^-3.90	7.8 × 10^–1^
J06-74	*hapA*	17.46-1.75 × 10^–8^	3.49 × 10^–6^	3.40 × 10^6^-3.40	6.8 × 10^–1^
J07-44	*hapA*	15.79-1.58 × 10^–8^	3.16 × 10^–4^	1.81 × 10^7^-1.81	3.6
L02-13	*hapA*	29.44-2.94 × 10^–8^	5.89 × 10^–3^	4.60 × 10^7^-4.60	9.2 × 10^1^
J09-62	*hapA*	10.63-1.06 × 10^–8^	2.13 × 10^–4^	6.20 × 10^6^-6.20	1.2 × 10^1^
L03-18	*hapA*	16.56-1.66 × 10^–8^	3.31 × 10^–5^	6.10 × 10^6^-6.10	1.2 × 10^1^
L10-04	*hapA*	12.69-1.27 × 10^–8^	2.54 × 10^–4^	1.25 × 10^7^-1.25	2.5 × 10^–1^
L10-05	*hapA*	13.82-1.38 × 10^–8^	2.76 × 10^–6^	3.00 × 10^6^-3.00	6.0 × 10^–1^
Q01-39	*hapA*	16.93-1.69 × 10^–8^	3.39 × 10^–4^	1.50 × 10^6^-1.50	3.0
Q04-43	*hapA*	12.37-1.24 × 10^–8^	2.47 × 10^–2^	5.60 × 10^8^-5.60	1.1 × 10^3^
Q06-10	*hapA*	36.50-3.65 × 10^–8^	7.30 × 10^–4^	1.36 × 10^7^-1.36	2.7
Q06-66	*hapA*	13.06-1.31 × 10^–8^	2.61 × 10^–3^	1.50 × 10^7^-1.50	3.0
b06-92	*hapA*	6.79-6.79 × 10^–9^	1.36 × 10^–2^	7.40 × 10^6^-7.40	1.5
b10-61	*hapA*	12.87-1.28 × 10^–8^	2.57 × 10^–3^	4.10 × 10^6^-4.10	8.2
b10-79	*hapA*	11.12-1.11 × 10^–8^	2.22 × 10^–3^	8.10 × 10^6^-8.10	1.6 × 10^1^
b11-89	*hapA*	9.18-9.18 × 10^–9^	1.84 × 10^–3^	4.90 × 10^6^-4.90	9.8
N03-06	*hapA*	12.49-1.25 × 10^–9^	2.50 × 10^–4^	7.20 × 10^6^-7.20	1.4
N04-21	*hapA*	6.50-6.50 × 10^–9^	1.30 × 10^–5^	1.53 × 10^7^-1.53	3.1
N04-58	*hapA*	16.76-1.68 × 10^–8^	3.35 × 10^–3^	9.60 × 10^6^-9.60	1.9
N04-70	*hapA*	6.61-6.61 × 10^–9^	1.32 × 10^–4^	6.50 × 10^6^-6.50	1.3 × 10^–1^
ATCC39315	*mshA*	48.11-4.81 × 10^–8^	9.62 × 10^–5^	–	–
B05-69	*mshA*	15.99-1.60 × 10^–8^	3.20 × 10^–6^	7.60 × 10^6^-7.60	1.5 × 10^–1^
Q07-21	*mshA*	14.07-1.41 × 10^–9^	3.65 × 10^–4^	1.09 × 10^8^-10.92	2.8 × 10^1^
L03-93	*mshA*	20.98-2.10 × 10^–8^	4.20 × 10^–4^	1.17 × 10^7^-1.17	2.3 × 10^1^
ATCC39315	*pilA*	48.11-4.81 × 10^–8^	9.62 × 10^–5^	–	–
J07-29	*pilA*	23.80-2.38 × 10^–8^	4.76 × 10^–3^	1.04 × 10^7^-1.04	2.1 × 10^–1^
J07-85	*pilA*	19.38-1.94 × 10^–8^	3.88 × 10^–5^	5.70 × 10^7^-5.70	1.1 × 10^–1^
L10-36	*pilA*	13.13-1.31 × 10^–9^	3.41 × 10^–4^	1.28 × 10^8^-12.77	3.3 × 10^1^
B01-01	*tlh*	12.13-1.21 × 10^–8^	2.91 × 10^–2^	1.32 × 10^7^-1.32	2.6 × 10^1^
B06-04	*tlh*	20.02-2.00 × 10^–8^	4.80 × 10^–3^	6.60 × 10^6^-6.60	1.3 × 10^1^
B06-69	*tlh*	21.11-2.12 × 10^–8^	3.46 × 10^–2^	1.63 × 10^7^-1.63	3.3 × 10^1^
B07-10	*tlh*	23.98-2.40 × 10^–8^	2.93 × 10^–3^	1.51 × 10^7^-1.51	3.0 × 10^1^
J02-13	*tlh*	9.55-9.55 × 10^–9^	1.48 × 10^–3^	8.90 × 10^6^-8.90	1.8 × 10^2^
J02-19	*tlh*	15.73-1.57 × 10^–8^	1.69 × 10^–2^	3.50 × 10^6^-3.50	7.0 × 10^1^
J02-24	*tlh*	15.06-1.51 × 10^–8^	3.01 × 10^–3^	13.1 × 10^6^-13.1	2.6 × 10^2^
J06-25	*tlh*	13.99-1.40 × 10^–8^	2.80 × 10^–4^	3.50 × 10^7^-3.50	7.0
J07-29	*tlh*	23.80-2.38 × 10^–8^	4.76 × 10^–4^	1.04 × 10^7^-1.04	2.1 × 10^–1^
L01-06	*tlh*	14.73-1.47 × 10^–8^	2.95 × 10^–3^	1.41 × 10^7^-1.41	2.8 × 10^1^
L02-20	*tlh*	17.18-1.72 × 10^–8^	4.00 × 10^–4^	3.90 × 10^6^-3.90	7.8 × 10^–1^
L03-10	*tlh*	12.59-1.26 × 10^–8^	4.24 × 10^–4^	1.14 × 10^7^-1.14	2.3
L10-06	*tlh*	11.21-1.12 × 10^–8^	2.24 × 10^–3^	8.60 × 10^6^-8.60	1.7 × 10^–1^
Q01-10	*tlh*	7.41-7.41 × 10^–9^	1.48 × 10^–3^	5.90 × 10^5^-5.90	1.2
Q01-35	*tlh*	17.30-1.73 × 10^–8^	3.46 × 10^–4^	1.01 × 10^7^-1.01	2.0 × 10^2^
Q04-55	*tlh*	14.63-1.46 × 10^–8^	3.15 × 10^–3^	9.40 × 10^6^-9.40	1.9
Q08-34	*tlh*	17.31-1.73 × 10^–8^	3.52 × 10^–2^	8.30 × 10^6^-8.30	1.7 × 10^–1^
Q10-54	*tlh*	8.47-8.47 × 10^–9^	1.69 × 10^–4^	5.90 × 10^5^-5.90	1.2
N05-39	*tlh*	17.58-1.76 × 10^–8^	3.08 × 10^–2^	1.47 × 10^7^-1.47	2.9
N05-77	*tlh*	14.54-1.45 × 10^–8^	3.30 × 10^–4^	1.18 × 10^7^-1.18	2.4
N06-55	*tlh*	15.38-1.54 × 10^–8^	1.91 × 10^–4^	1.33 × 10^7^-1.33	2.7
N08-74	*tlh*	16.49-1.65 × 10^–8^	3.15 × 10^–2^	1.29 × 10^6^-1.29	2.6 × 10^–1^
GIM 1.449	*tlh*	14.80-1.48 × 10^–9^	3.90 × 10^–3^	1.44 × 10^6^-14.38	3.7 × 10^–1^
ATCC39315	*tlh*	48.11-4.81 × 10^–8^	9.62 × 10^–3^	–	–

*−, not detected. ^∗^: the LOD values were obtained according to the genomic DNA dilutions or cell culture dilutions used in this study.*

**FIGURE 5:**
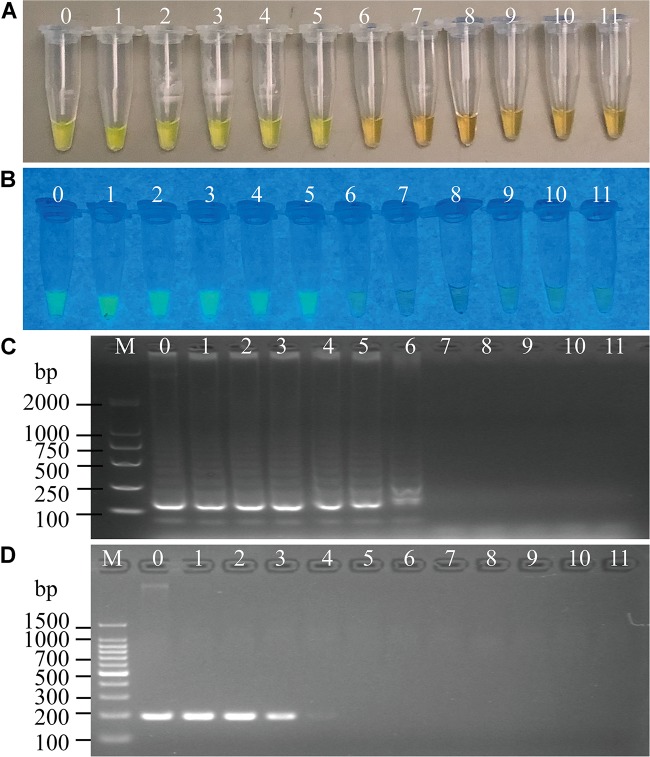
Sensitivity of the sssvLAMP method for the detection of the *mshA* gene of *V. cholerae* genomic DNA. **(A–C)** The results observed by the naked eye under the visible light **(A)** and the UV light (302 nm) **(B)**, and verified by 2% agarose gel electrophoresis analysis **(C)** by the sssvLAMP method. **(D)** The results by the PCR assay. Lane M: DNA molecular weight Marker (100 bp). Tubes/Lanes 0–10: containing 14.07 ng/μL –14.07 × 10^–10^ ng/μL of *V. cholerae* Q07-21 genomic DNA; 11: negative control.

The LOD of the sssvLAMP method for genomic DNA of the 52 *V. cholerae* strains was also determined. For instance, for the target gene *hapA*, genomic DNA ranged from 6.50 to 9.18 × 10^–9^ ng/μL of 24 *V. cholerae* (*hapA*^+^) strains was tested, and the results indicated that the sensitivity of the sssvLAMP method ranged from 1.36 × 10^–2^ to 4.42 × 10^–6^ ng/reaction (Figures not shown). Similarly, for the target genes *mshA*, *pilA*, and *tlh*, the LOD values ranged from 3.65 × 10^–4^ to 3.20 × 10^–6^ ng/reaction, from 4.76 × 10^–3^ to 9.62 × 10^–5^ ng/reaction, and from 1.69 × 10^–2^ to 4.76 × 10^–4^ ng/reaction, respectively, when 3.20-1.41 × 10^–9^ ng/μL, 13.13-4.81 × 10^–8^ ng/μL, and 8.47-9.55 × 10^–9^ ng/μL genomic DNA samples of *V. cholerae* strains (*mshA*^+^, *pilA*^+^, and *tlh*^+^) were tested in the sssvLAMP method, respectively (e.g., Figure 5) (Table 5). These results also indicated strain-dependent variation in LOD (CFU versus genomic DNA of *V. cholerae*).

Comparison of the sensitivity by the routine PCR assay. For example, as shown in Figure 4D, the LOD of *hapA* gene by the PCR assay was 6.0 × 10^4^CFU/mL and 1.1 × 10^–3^ ng DNA/μL for the detection of *V. cholerae* GIM 1.449 cells and genomic DNA samples, respectively, which indicated that the sssvLAMP method was 6.45 × 10^4^-, and 6.50 × 10^3^-fold more sensitive than the PCR assay. Similarly, for the target gene *mshA* in *V. cholerae* Q07-21, our data showed the LOD of 7.10 × 10^5^ CFU/mL, and 9.15 ng DNA/μL by the PCR, which were 650.18, and 650.32-fold lower than the sssvLAMP method (Figures not shown). Likewise, the LOD of the *pilA* gene in *V. cholerae* L10-36 by the PCR assay was 8.30 × 10^4^ CFU/mL, and 8.53 × 10^–1^ ng DNA/μL, respectively, which were 65-fold lower than LAMP assays, while those of the *tlh* gene in *V. cholerae* GIM 1.449 were 9.35 × 10^4^CFU/mL, and 9.62 × 10^–1^ ng DNA/μL respectively, indicating that the sssvLAMP method was 6.50 × 10^3^, and 6.41 × 10^3^-fold more sensitive than the PCR assay, respectively (e.g., Figure 5).

Taken together, for the detection of *V. cholerae* cells and genomic DNA samples in water, the sensitivity of the sssvLAMP method for the virulence-associated genes were 6.50 × 10^1^-6.45 × 10^4^, and 6.50 × 10^1^-6.50 × 10^3^-fold sensitive than the PCR assay for these tested *V. cholerae* strains, respectively.

### Sensitivity of the sssvLAMP Method for Spiked Fish, Shrimp and Shellfish Samples, as Well as Human Stool Specimens

**FIGURE 6:**
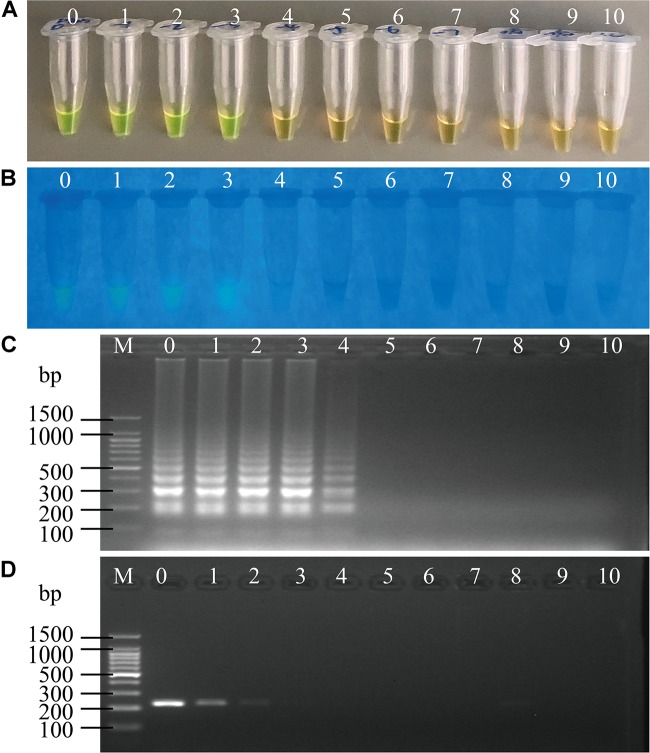
Sensitivity of the sssvLAMP method for the detection of the *pilA* gene in the spiked fish matrix samples. **(A–C)** the results observed by the naked eye under the visible light **(A)** and the UV light (302 nm) **(B)**, and verified by 2% agarose gel electrophoresis analysis **(C)** by the sssvLAMP method. **(D)** The results by the PCR assay. Lane M: DNA molecular weight Marker (100 bp). Tubes/Lanes 0–9: 4.85 × 10^6^-4.85 × 10^–3^ CFU/Ml of *Vibrio cholerae* L10-36 cells; 10: negative control.

Cell cultures of *V. cholerae* strains GIM1.449, Q07-21 and L10-36 were added into *P*. *pekinensis* homogenates, respectively, and the sensitivity of the sssvLAMP Method was determined. The observed LOD values ranged from 0.2 to 250 CFU/reaction of *V. cholerae*, which were 6.5 × 10^3,^ 6.5 × 10^2^, 11.50, and 6.5 × 10^2^-fold higher than those of the PCR assay for targeting the *hapA*, *mshA*, *pilA*, and *tlh* genes, respectively (e.g., Figure 6). Additionally, each of the three strains was spiked into *L. vannamei*, and *P. viridis* homogenates, respectively, and LOD values for the four target genes were evaluated. The resulting data showed that the sssvLAMP Method was more sensitive for the *L. vannamei* matrix (LOD: 0.59–8.7 CFU/reaction) than for the *P. viridis* matrix (LOD: 8.7–590 CFU/reaction) (Table 6). Additionally, health human stool specimens were spiked with each of the *V. cholerae* GIM1.449, Q07-21 and L10-36 strains, and the sensitivity of the sssvLAMP method was also tested. As shown in Table 6, the LOD values ranged from 0.59 to 87 CFU/reaction targeting the four virulence-associated genes tested, suggesting application potential in the detection of human clinical diarrhea patient samples in the future.

**TABLE 6:** Sensitivity of the sssvLAMP method for spiked aquatic product samples and human stool specimens.

**Matrix sample**	**Spiked *V. cholerae* strain**	**Cell culture dilutions (CFU/mL)**	**Target gene**	**LOD^∗^/reaction (CFU)**
*P. pekinensis*	GIM1.449	9.80 × 10^8^-9.80	*hapA*	2.0 × 10^–1^
*L. vannamei*	GIM1.449	11.8 × 10^7^-11.8	*hapA*	5.9
*P. viridis*	GIM1.449	11.8 × 10^7^-11.8	*hapA*	5.9 × 10^1^
Human stool	GIM1.449	11.8 × 10^7^-11.8	*hapA*	5.9
*P. pekinensis*	L10-36	1.27 × 10^9^-1.27	*pilA*	2.5 × 10^2^
*L. vannamei*	L10-36	17.4 × 10^7^-17.4	*pilA*	8.7
*P. viridis*	L10-36	17.4 × 10^7^-17.4	*pilA*	8.7
Human stool	L10-36	17.4 × 10^7^-17.4	*pilA*	8.7 × 10^1^
*P. pekinensis*	Q07-21	6.30 × 10^8^-6.30	*mshA*	1.3 × 10^2^
*L. vannamei*	Q07-21	14.3 × 10^7^-14.3	*mshA*	7.2
*P. viridis*	Q07-21	14.3 × 10^7^-14.3	*mshA*	7.2 × 10^1^
Human stool	Q07-21	14.3 × 10^7^-14.3	*mshA*	7.2 × 10^1^
*P. pekinensis*	GIM1.449	9.80 × 10^8^-9.80	*tlh*	2.0 × 10^2^
*L. vannamei*	GIM1.449	11.8 × 10^7^-11.8	*tlh*	5.9 × 10^–1^
*P. viridis*	GIM1.449	11.8 × 10^7^-11.8	*tlh*	5.9 × 10^2^
Human stool	GIM1.449	11.8 × 10^7^-11.8	*tlh*	5.9 × 10^–1^

*^∗^, the LOD values were obtained according to the genomic DNA dilutions or cell culture dilutions used in this study.*

**FIGURE 7:**
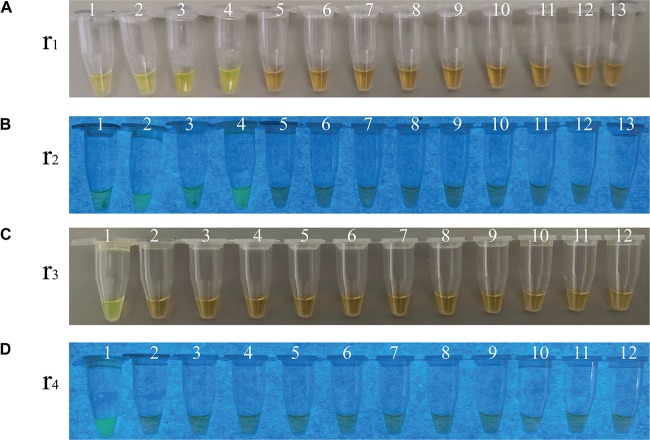
Specificity of the sssvLAMP method for the detection of the *ctxA*, *tcpA, lolB*, and *hapA* genes in some bacterial strains. **(A–D)** The results observed by the naked eye under the visible light **(A,C)** and the UV light (302 nm) **(B,D)**. Tubes 1–13 **(A,B)**: containing genomic DNA of *V. cholerae* ATCC39315 (N16961); *V. cholerae* ATCC39315 (N16961); *V. cholerae* ATCC39315 (N16961); *V. cholerae* GIM1.449; *V. fluvialis* ATCC33809; *V. harvey* ATCC BAA-1117; *V. harveyi* ATCC33842; *V. mimicus* bio-56759; *V. metschnikovii* ATCC 700040; *V. parahemolyticus* ATCC17802; *V. parahemolyticus* ATCC33847; *V. vulnificus* ATCC27562; and negative control, respectively. Tubes 1-12 **(C,D)**: containing genomic DNA of *V. cholerae* GIM1.449 (*lolB*); *A. hydrophila* ATCC35654; *E. cloacae* ATCC13047; *E. coli* ATCC8739; *E. sakazakii* CMCC45401; *L. monocytogenes* ATCC19115; *P. aeruginosa* ATCC9027; *S. enterica* subsp. Enterica Leminor et popoff ATCC13312; *S. typhimurium* ATCC15611; *S. flexneri* ATCC12022; *V. alginolyticus* ATCC33787; and negative control, respectively. All the tubes contained the LAMP primers targeting the *hapA* gene, except the tubes 1 and 2 **(A,B)**, and tube 1 **(C,D)** targeting the *ctxA*, *tcpA*, and *lolB* genes, respectively. The highest genomic DNA concentrations given in Table 5 were used in the specificity assays.

### Reproducibility of the sssvLAMP Method

For all target genes, all positive results were repeated in all the experiments performed not only for *V. cholerae* cells or genomic DNA samples, but also for the spiked fish samples within the LOD ranges, indicating high reproductivity (100%) of the sssvLAMP method developed in this study (Figures not shown).

### Detection of Water and Fish Samples by the sssvLAMP Method

Eighteen water samples collected from various sources in June of 2019 in Shanghai, China were examined by the sssvLAMP method for the target genes of *V. cholerae*. As shown in Table 4, all the water samples were negative for the targeted genes, indicating the drinking water and environmental water samples tested were negative for potential pathogenic *V. cholerae*. Additionally, four commonly consumed fish species samples (*A. nobilis*, *C. auratus*, *C. idellus*, and *P. pekinensis*) were also examined by the sssvLAMP method. The results showed that all the fish meat samples were negative for the targeted genes. However, among the twelve fish intestinal samples, seven were positive for *hapA, pilA*, and/or *tlh* genes. Although only a small number of samples were detected, variable virulence-associated gene profiles were observed among intestinal samples of the fish species (Table 4). The *hapA* and *tlh* genes were detected in the *A. nobilis* intestinal samples, while the former gene also existed in the *C. auratus* and *P. pekinensis* intestines. The *pilA* gene was only present in *P. pekinensis* intestines, whereas all the virulence-associated genes were absent from *C. idellus* intestinal samples. The results were confirmed by the PCR assay and the conventional isolation and identification assay of *V. cholerae* from the fish intestinal samples.

## Discussion

The development of rapid, sensitive, and cost-effective methods is needed not only for the quick and appropriate treatment of patients infected with pathogenic *Vibrio* strains, but also for the prompt control and prevention of the diseases (Okada et al., 2010). To date, only few studies have been conducted on the detection of *V. cholerae* genes by the LAMP technique (He et al., 2009; Okada et al., 2010; Srisuk et al., 2010; Tourlousse et al., 2012; Liew et al., 2015; Zhang et al., 2015). In this study, an sssvLAMP method was successfully developed for the detection of virulence-associated genes *ctxA*, *tcpA*, *hapA*, *mshA*, *pilA*, and *tlh* of *V. cholerae*, as well as species-specific gene *lolB* of the bacterium.

The magnesium is required for the LAMP reaction, however, the lower or higher concentration of Mg^2+^ strongly influenced the LAMP reaction and its product. For instance, the excessive Mg^2+^ stabilizes the mispriming of primers that decrease the specificity of the assay, and also prevents the denaturation of the DNA double strands by stabilizing the duplex structure (Markoulatos et al., 2002). In contrast, lower concentrations of Mg^2+^ fail to amplify the target genes. In this study, the optimal concentration of Mg^2+^ was 6.0 mM for the sssvLAMP method, which was consistent with previous studies on the detection of *ctxA* gene of *V. cholerae* (Engku Nur Syafirah et al., 2018), dengue viruses (Kim et al., 2018), and *Mycoplasma hyopneumoniae* (Liu et al., 2015), but less than those (8 mM) for the detection of *ctxA* and *ompW* genes of *V. cholerae* (Okada et al., 2010; Srisuk et al., 2010).

The dNTP is essential for the synthesis of double-stranded DNAs in the nucleic acid amplification *in vitro*. In this study, the LAMP amplification was observed in all reaction tubes containing 1.0–2.0 mM dNTP, and the concentration of 1.0 mM was chosen for the sssvLAMP method, which was less than some previous reports (Goto et al., 2009; Srisuk et al., 2010). The minimum usage of dNTP reduces the cost of the method, particularly when a large number of samples need to be analyzed.

In the LAMP system, the autocycling and strand displacement of DNA synthesis were mediated by the *Bst* DNA polymerase and four specially designed primers (Notomi et al., 2000). It has been reported that the LAMP reaction could be accelerated by the addition of the loop primers (Nagamine et al., 2002; Lim et al., 2013). In this study, our results indicated that positive amplicons were initially detected at a reaction time of 30 min with the loop primers, which was 20-min faster than those without the loop primers. The significance of optimal concentrations of each pair of primers in the LAMP system has been pointed out in previous studies (Okada et al., 2010; Srisuk et al., 2010; Lau et al., 2011; Kakizaki et al., 2018), in which a 1.6 μM of the inner primers, 0.2 μM of the outer primers, and 0.8 μM of the loop primers was often used with an optimal ratio 8:1:4 (μM/μM/μM) of the three primers. In this study, our data showed that 1.60 μM of inner primers, 0.05 μM of the outer primers, and 0.2 μM of the loop primers were optimum for the sssvLAMP method with a final ratio of 32:1:4 (μM/μM/μM), showing fourfold less amount of the outer and loop primers. The elongation reactions in the LAMP system are sequentially repeated by *Bst* DNA polymerase-mediated strand-displacement synthesis (Notomi et al., 2000, 2015). In this study, an 8 U concentration of *Bst* DNA polymerase was used in the sssvLAMP reaction, which yielded positive amplicons with the distinct color change, consistent with some previous studies (Priya et al., 2018; Huang et al., 2019; Zhang and Gleason, 2019), but higher than the other assays (Srisuk et al., 2010; Yamazaki, 2011).

In this study, all positive reactions can be easily judged by the naked eye under the visible light, or under the UV light by the use of MnCl_2_-calcein dye in the LAMP system prior to amplification. Moreover, the sssvLAMP method did not require opening of reaction tubes with no probable cross-contamination, which usually arise from opened tubes after amplification (Sayad et al., 2018). Furthermore, the sssvLAMP method was carried out in a one-step reaction at 65°C for 40–50 min, which was faster than the PCR assay and more suitable for low-equipment setting laboratories.

Sensitivity is particularly important in the detection of foodborne pathogenic bacteria (Yuan et al., 2018). It has been reported that 10 CFU per reaction targeting bacterial resistance integrons (*intI1*, *intI2*, and *intI3*) (Yu et al., 2014), 8 and 0.54 CFU per reaction targeting the *ompW* (Srisuk et al., 2010) and *ctxA* (Okada et al., 2010) genes of *V. cholerae*, respectively, could be detected by LAMP methods. In this study, for the target genes *hapA*, *mshA*, *pilA*, and *tlh*, the LOD values ranged from 9.2 × 10^1^ to 1.1 × 10^–1^CFU/reaction for the most of 52 *V. cholerae* strains in the inclusivity tests. Given the LOD values below 1 CFU/reaction, one possibility could be non-culturable *Vibrio* cells in cell dilutions. Another possibility could be the released genomic DNA from cracked *V. cholerae* cells during cell incubation, or from thermal lysis reactions before the sssvLAMP assays. These DNA in dilutions could be detected by the sssvLAMP method. Tourlousse et al. (2012) reported that detection and quantification of 10–100 genomes per μL could be performed in a polymer microfluidic chip by a real-time fluorogenic LAMP for targeting *rtxA* and *toxR* genes of *V. cholerae*. Different sensitivities of the LAMP technique were also reported, e.g., 6.2 pg DNA per tube for the *hlyA* (He et al., 2009) and 5 fg DNA per reaction for the *ctxA* (Okada et al., 2010) genes of *V. cholerae*, 100 pg DNA per μL for resistance gene *vanA* of *Enterococcus faecium* (Huang et al., 2019), and 10 pg DNA per tube for bacterial resistance integrons (*intI1*, *intI2*, and *intI3*) (Yu et al., 2014), all of which were less sensitive than those of the *hapA* (4.42 × 10^–6^ ng DNA/reaction) and *tlh* (3.90 × 10^–3^ ng DNA/reaction) genes of *V. cholerae* GIM1.449 by the sssvLAMP method developed in this study (Table 5). Variable LOD for the same or different targeted genes in different strains of *V. cholerae* may be related to the amplification efficiency of designed primers, or the multiple occurrence of a toxigenic gene in the genome of a particular stain, which may be different than that of another strain harboring the same gene. Additionally, variable sensitivity of the sssvLAMP method was observed when it was used to detect aquatic product samples and human stool specimens, which may be resulted from the influence of different matrix in homogenate samples tested.

The specificity data of the sssvLAMP method indicated that the method were applicable only in the existence of both the inner and outer primers. Moreover, no false positive amplification of the targeted genes of *V. cholerae* was observed in the closely related *Vibrio* species, as well as in non-targeting pathogenic bacteria tested, indicating high specificity of the designed primers targeting virulence-associated genes of *V. cholerae*. High occurrence of *V. cholerae* isolates carrying target genes has been reported (Meena et al., 2019; Xu et al., 2019), e.g., *hapA* (95.0%), and *tlh* (76.0%) in the fish species (Xu et al., 2019). Since the target genes can be present in many non-pathogenic strains of *V. cholerae*, cytotoxicity or enterotoxicity experiments may be performed to further justify pathogenesis of target genes-positive *V. cholerae* strains.

In this study, similar high efficiency of the sssvLAMP method was observed when tested with spiked samples of water and aquatic products, as well as human stool specimens. The results showed none of the *V. cholerae* contamination in all drinking water and environmental water samples, as well as human stool specimens tested. However, virulence-associated gene Types IV, V, and VII were found from the intestinal samples from three of the four commonly consumed fish species in China, including *A. nobilis*, *C. auratus*, and *P. pekinensis*, in which *hapA*, *tlh*, and *pilA* genes were detected positive. High occurrence of the *hapA* gene was also detected in the presence of *V. cholerae* strains isolated from the fish intestines (Xu et al., 2019), which may be related to its function involved in *V. cholerae* interaction with aquatic hosts (Halpern et al., 2003). In contrast, the *mshA* gene was absent from the fish intestinal samples, and low percentage was present in the 52 *V. cholerae* strains tested in this study, suggesting missing or truncation of the *mshA* gene in the bacterium, although the MSHA gene cluster is reported to exist and aid bacterial association with aquatic plankton to support environmental adaptation in many non-O1/O139 strains (Chiavelli et al., 2001; Moorthy and Watnick, 2004; Gong et al., 2019). The finding in this study, coupled with the previous research enhanced need for regular monitoring of *V. cholerae* contamination in these aquatic products for ensuring food safety.

Overall, the sssvLAMP method developed in this study was simple, rapid, and visible to the naked eye, showing greater advantages when compared with the routine PCR assay. This method was successfully employed to detect virulence-associated genes *hapA*, *mshA*, *pilA* and *tlh*, toxigenic gene *ctxA* and *tcpA*, and species-specific gene *lolB* of *V. cholerae*. In the future research, the sssvLAMP method should detect other important virulence factors, e.g., Type III secretion system, non-agglutinable heat-stable enterotoxin (NAG-ST), and cholix toxin, which have been shown to induce entertoxicity or cytotoxicity. It should support the field or clinical diagnosis where rapid and reliable detection of virulence-associated genes of *V. cholerae* is urgently required.

## Data Availability Statement

The datasets generated for this study are available on request to the corresponding author.

## Author Contributions

MX, ZS, JZ, WA, and LC participated in the design and or discussion of the study. MX, HF, and DC carried out the experiments. MX, HF, and LC analyzed the data. MX and LC wrote the manuscript. WA revised the manuscript. All authors read and approved the final version to be published.

## Conflict of Interest

The authors declare that the research was conducted in the absence of any commercial or financial relationships that could be construed as a potential conflict of interest.

## Footnotes

1 http://www.who.int/

2 https://www.ncbi.nlm.nih.gov/genbank

3 http://primerexplorer.jp/lampv5e/index.html

## Supplementary Material

FIGURE S1 Comparative sequence alignments between the newly designed LAMP primers versus the targeted genes *hapA* **(A)**, *lolB* **(B)**, *mshA* **(C)**, *pilA* **(D)**, *tlh* **(E)**, and *tcpA* **(F)** amplified in representative strains by PCR reactions with GenBank accession numbers MN708522 to MN708551.
