# Supplementary figures and images for: Simple Visualized Detection Method of Virulence-Associated Genes of Vibrio cholerae by Loop-Mediated Isothermal Amplification
Source: Front Microbiol. 2019 Dec 20;10:2899. doi: 10.3389/fmicb.2019.02899 (PMC6932958; doi:10.3389/fmicb.2019.02899)

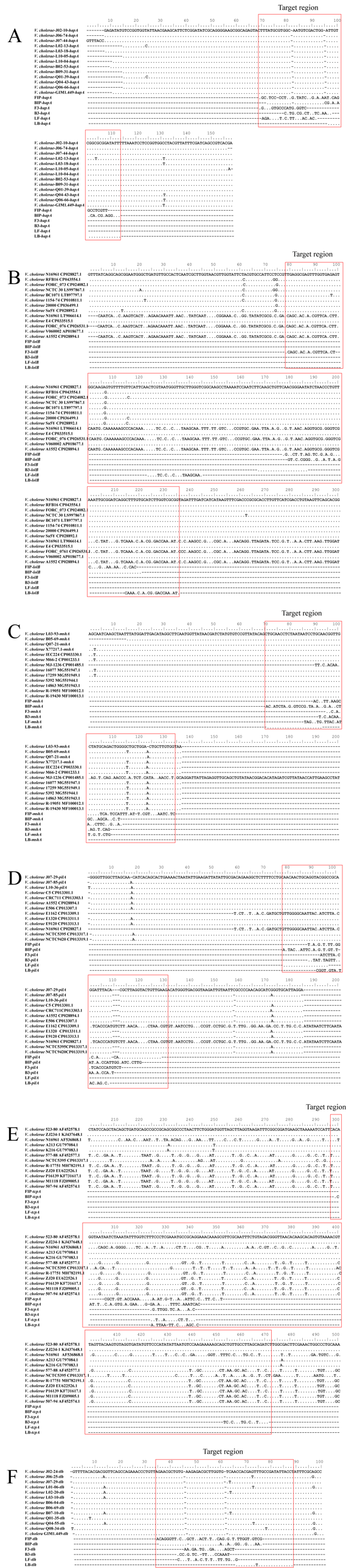

Supplement: FIGURE S1 — Comparative sequence alignments between the newly designed LAMP primers versus the targeted genes hapA (A), lolB (B), mshA (C), pilA (D), tlh (E), and tcpA (F) amplified in representative strains by PCR reactions with GenBank accession numbers MN708522 to MN708551. [file Image_1.TIF]
